# Interactions between a Polygenic Risk Score and Non-genetic Risk Factors in Young-Onset Breast Cancer

**DOI:** 10.1038/s41598-020-60032-3

**Published:** 2020-02-24

**Authors:** M. Shi, K. M. O’Brien, C. R. Weinberg

**Affiliations:** 1 Biostatistics and Computational Biology Branch, National Institute of Environmental Health Sciences, Research Triangle Park, North Carolina, United States; 2 Epidemiology Branch, National Institute of Environmental Health Sciences, Research Triangle Park, North Carolina, United States

**Keywords:** Breast cancer, Genetic association study, Risk factors

## Abstract

Most gene-environmental studies have focused on breast cancers generally, the preponderance of which occur after age 50. Young-onset breast cancers (YOBC) tend to be aggressive and may be etiologically different. The goal of this analysis was to assess interactions between an established 77-SNP polygenic risk score (PRS) and non-genetic risk factors for YOBC. We constructed the PRS using a family-based study of 1,291 women diagnosed with breast cancer before age 50 and their parents and unaffected sisters. We used conditional logistic regression to analyze interactions between the PRS and 14 established risk factors. In further analyses we assessed the same interactions, but for invasive cancer, estrogen receptor (ER) positive cancer and with broader inclusion of racial/ethnic groups. Results showed a decreased association between the PRS and YOBC risk for women who had ever used hormonal birth control (odds ratio [OR] = 2.20 versus 3.89) and a stronger association between the PRS and YOBC risk in pre-menopausal women (OR = 2.46 versus 1.23). Restricting the analysis to ER+ cancers or invasive cancers or using samples from all ethnic groups produced similar results. In conclusion, the PRS may interact with hormonal birth control use and with menopausal status on risk of YOBC.

## Introduction

Breast cancer is the most common cancer in women, with an estimated 268,600 new cases in the U.S. in 2019 (https://www.cancer.org/content/dam/cancer-org/research/cancer-facts-and-statistics/annual-cancer-facts-and-figures/2019/cancer-facts-and-figures-2019.pdf). Genetic variants contributing to breast cancer risk range from rare variants with moderate to high penetrance to common variants with low penetrance, and GWAS studies have identified hundreds of genetic risk variants^1^. Many of these variants are also identified in studies of young-onset breast cancer^2,3^, which tends to be more aggressive, but some may be predictive of young-onset disease, specifically^2,4^. In terms of reproductive and behavioral risk factors, overall breast cancer risk is associated with early age at menarche, late age at first birth, low parity, late age at menopause, hormone replacement therapy, alcohol consumption, recent oral contraceptive use, and low physical activity (https://www.cdc.gov/cancer/breast/basic_info/risk_factors.htm). High body mass index (BMI) is an established risk factor for postmenopausal breast cancer, but has been shown to be inversely associated with premenopausal breast cancer^5^, another indication of etiologic differences between earlier and later-onset disease.

Gene-environment interactions can be important to identify, both because they offer clues about etiology and because they can ultimately help us build algorithms through which we can distinguish high risk from low risk women and devise more personalized screening plans based on well-informed risk assessment. Gene-environment interaction effects for overall breast cancer have been assessed by a number of studies, most focusing on the established genetic and reproductive/behavioral risk factors. Overall, the results suggest that the interaction effects are weak^6–15^. Recently Rudolph *et al*. studied interactions between the 77-SNP polygenic risk score (PRS) that was originally proposed by Mavaddat^16^ and 7 epidemiological factors, using data from the Breast Cancer Association Consortium, and identified interaction between PRS and alcohol consumption, height and hormone therapy^17^. Case-control studies of gene-by-environment interaction can be vulnerable to exposure-related population stratification if the source population includes subpopulations with differing exposure prevalences, which also differ in their LD between genetic markers and causative variants^18^. While not fully robust against such bias, family-based studies are far less vulnerable to such effects, because they implicitly match on ancestry. (Figure 1 of Weinberg *et al*.^18^). In this paper we assess interactions between the same 77-SNP PRS^3^ and 14 established risk factors for breast cancer using data from a family-based genome-wide association study (GWAS) of young-onset (under age 50) breast cancer, the Two Sister Study.

## Methods

### Study sample

Families were recruited through the Sister Study and the Two Sister Study, as described previously^4^. Briefly, the Sister Study is a prospective breast cancer study that enrolled women (2003–2009) who had one or more sisters diagnosed with breast cancer but had never had breast cancer themselves. Families where the proband sister had been diagnosed with invasive breast cancer or ductal carcinoma *in situ* (DCIS) before age 50 and within the previous four years were eligible to participate in the ancillary Two Sister Study (2008–2010). The proband case sister as well as any living parents were invited to participate by providing DNA samples. Both the proband case and her breast cancer-free (control) sister completed the same computer-assisted telephone interviews about various health-related and lifestyle factors and Sister Study participants provided blood samples. This genetic substudy included Two Sister Study participants and their parents, and some Sister Study participants who developed incident breast cancer (invasive or DCIS) before age 50 during the follow-up period. All participants provided written informed consent in participating in this study and the study was approved by the National Institute of Environmental Health Sciences and the Copernicus Group Institutional Review Boards and all research was performed in accordance with relevant guidelines/regulations. We note that while the Sister Study is enriched for family history, the add-on Two Sister Study did not require having any breast cancer family history prior to the recruited case.

### Risk factor assessment

We limited our main analysis to the main ethnic subset, i.e. non-Hispanic white women, and assessed interactions with the following risk factors: height (continuous; cm), self-reported BMI at 30 (premenopausal women only), cigarette smoking, alcohol, menopausal status, age at menarche, hormonal birth control use, years of using hormonal birth control, recency of hormonal birth control use (within 5 years), parity, age at first birth, recency of last birth (within 5 years), and breastfeeding (among parous women). We did not include hormone replacement therapy use due to concerns about the abrupt historical shifts over time in the prevalence of use and systematic age differences between cases and controls^19^. To ensure comparable opportunities for exposure between the matched sisters, the time-dependent risk factors that we did assess (e.g. menopausal status) were defined as of the proband’s age at breast cancer diagnosis. In other words, risk factor changes that had been experienced by an older control sister at ages after the age at which her case sister had been diagnosed were not considered relevant. All controls had aged past that age without a diagnosis of breast cancer. We used follow-up data from the Sister Study to fill in risk factor information for controls who had been enrolled at an age younger than their case sister’s diagnosis age.

The genotype data were obtained from a previous GWAS study (http://www.ncbi.nlm.nih.gov/projects/gap/cgi-bin/study.cgi?study_id=phs000678.v1.p1)^4^. To construct the multiplicative 77-SNP polygenic risk score (PRS), originally proposed by Mavaddat *et al*.^3,16^ we used the natural log of the product of the 77 risk-allele-specific odds ratios (based on the number of copies of the risk allele) to calculate the PRS. We used the published ORs by Mavaddat (2015) in the calculation. Forty of the 77 SNPs were directly genotyped on the Illumina OmniExpress plus HumanExome-8v1-2 arrays used in the GWAS, and for the remaining 37 non-genotyped SNPs we used imputed genotypes. All 40 genotyped SNPs had a high call rate (≥97%) and low discordance rate (≤1%) in 74 study duplicates. “Impute” (v2.3.0) was used to impute the genotypes and the average quality score for the 37 imputed SNPs was 0.99. Details of the risk score calculation are given in Shi *et al*.^3^. Individual SNP association results are shown in Table 3 of Shi *et al*.^3^. Even though these SNPs were GWAS hits mainly for older onset breast cancer, we have shown previously that many of them were also associated with young-onset breast cancer^3^ and the 77-SNP PRS is a reasonable measure for young-onset breast cancer.

### Outcome definitions

Breast cancer cases were self-reported, with high confirmation rates seen for those who also provided medical records^20^. We extracted tumor information including invasiveness, and estrogen receptor (ER) status from the medical record for most of the women (95% to 97%) and relied on self-report for the remainder. Controls in our study are either the actual sisters who were not diagnosed with breast cancer or the “pseudo-sister” when parents but not the unaffected sister were genotyped. The pseudo-sister is a hypothetical (but equally likely) sister who carries the parental alleles that were not transmitted to the case sister and has the same risk factors as those experienced by the actual unaffected sister^21^. We assume for this analysis that, conditional on the parents, the alleles that were transmitted do not influence the daughter’s status with respect to the risk factors under study.

### Statistical methods

Based on the availability of genotype and risk factor data, families in our study fall into four disjoint groups: case-parents-plus, case-parents, case-sibling-plus and case-plus. All of these families provided both risk factor and genotype data for the cases. The “case”, “parents” or “sibling” in the first part of the name denote that genotype data are available for the corresponding individuals, and the “plus” in the name denotes that data on the control sister’s risk factors was also available. Among non-Hispanic white families, we had 384 case-parents-plus families, 32 case-parents families, 432 case-sibling-plus families and 304 case-plus families.

We used conditional logistic regression (CLR) to take advantage of the matched design and enforce within-family gene-by-risk-factor independence^21^. This is a weak independence assumption, which only asserts that genetic differences between full sisters do not correspond to differences between the studied nongenetic factors in any systematic way. Conditional on the parents, the alleles that happened to be transmitted are not associated with the risk factors. The matching sets used in CLR for the four family structures in our data are summarized in Table 1.

**Table 1:** Four families structures.

Study Design	Risk Factor		Genotype			Matching Ratio	Matching Set^a^	N
	Case	Sibling	Case	Sibling	Both Parents			
Case-parents-plus	X	X	X		X	1:3	(Gc,Ec), (Gps, Es), (Gc, Es), (Gps, Ec)	384
Case-parents	X		X		X	1:1	(Gc,Ec), (Gps, Ec)	32
Case-sibling-plus	X	X	X	X		1:3	(Gc,Ec), (Gs, Es), (Gc, Es), (Gs, Ec)	432
Case-plus	X	X	X			1:1	(Gc,Ec), (Gc, Es)	304

“X” in the table denote the corresponding data are available.

^a^Gc, Gs and Gps denote the genotypes of the case, sibling and pseudo-sibling (see text) respectively. Ec and Es denote the exposure (risk factor) of the case and sibling respectively.

More specifically, for case-parents-plus and case-sibling-plus families, each case was matched with 3 other hypothetical genotype/risk-factor combinations: (1) her sister’s actual genotypes and risk factors; (2) her own genotypes and her sister’s risk factors and (3) her sister’s genotypes and her own risk factors. Note that here the genotype for the “sister” denotes either the actual control sister in the family or the pseudo-sister as defined above. Case-parents families have a 1:1 matching: each case’s genotypes and risk factors are matched with the pseudo-sister’s genotypes and the case’s risk factors. Case-plus families also have a 1:1 matching with the case’s genotypes and her sister’s risk factors.

We estimated risk factor marginal effects as well as multiplicative gene-by-risk-factor interaction effects. For all models, we adjusted for birth order to correct for the fact that because of the sampling design, control sisters were more often older than their case sister^22^. We implicitly matched on age by assessing risk factors for both sisters as of the case’s age at diagnosis. In addition, we adjusted for potential confounders as follows: parity and age at first birth when estimating the effects of breastfeeding, for alcohol when estimating the effects of smoking, for smoking when estimating the effects of alcohol, for age at menarche when estimating the effects of years using hormonal birth control, and for age at first birth when estimating the effects of parity (using parous women). All categorical variables except smoking have a natural ordering, and for those we assumed a linear trend in the interaction term but still treated the risk factor as categorical for its main effects. This ensured that the models were saturated for the risk factor main effect and thus correctly specified under a no-interaction null scenario^23^. In models targeting PRS-risk-factor interactions, we centered PRS of all individuals by subtracting the mean PRS based on the control sisters. With this centering, the estimated non-genetic risk factor main effect corresponds to the estimated effect when PRS is at the mean of the controls.

We performed the main analysis among non-Hispanic white breast cancer families, then further restricted to families with invasive or to families with ER+ breast cancer to examine whether any effect estimates changed substantially. To make use of all available data, we also conducted analyses using samples that included all racial/ethnic groups.

## Results

The study included risk factor data for a total of 1152 non-Hispanic white cases and their families, which included 1120 controls from the Sister Study. The case sisters had a higher average PRS (mean of 5.13, SD of 0.44) than control sisters (mean of 5.01, SD of 0.46). The average age at diagnosis of breast cancer was 45. Most of the breast cancers were invasive (85%) and most were ER+ (81%). The distributions of the risk factors in the case and control sisters in non-Hispanic white families are shown in Table 2. Supplemental Table 1 shows the distributions among all families in this study.

**Table 2:** Characteristics of the non-Hispanic white participants.

Variable	Case (N = 1152)	Control (N = 1120)
PRS mean (SD)	5.13 (0.44)	5.01 (0.46)
Age at Diagnosis mean (SD)	44.9 (3.9)	
Height mean (SD)	65.1 (2.5)	65 (2.6)
BMI at 30 s among Pre-menopausal mean (SD)	23.5 (3.9)	23.8 (4.4)
Age at Menarche mean (SD)	12.7 (1.5)	12.8 (1.5)
Age at First Birth as Continuous mean (SD)	27.6 (5.3)	26.7 (5.2)
**Invasive**		
Yes	984 (0.85)	
No	168 (0.15)	
**ER+**		
Yes	917 (0.81)	
No	222 (0.19)	
**Smoking**		
Never	748 (0.65)	731 (0.65)
Former	319 (0.28)	286 (0.26)
Current	85 (0.07)	102 (0.09)
**Alcohol**		
Never	121 (0.11)	88 (0.08)
<1 drink/day	896 (0.78)	896 (0.81)
≥1 drink/day	131 (0.11)	118 (0.11)
**Pre-menopausal status as of index age**		
Yes	1069 (0.93)	999 (0.89)
No	81 (0.07)	120 (0.11)
**Hormonal birth control use**		
Yes	1046 (0.91)	997 (0.89)
No	102 (0.09)	121 (0.11)
**Years using hormonal birth control prior to Index age**		
<2	259 (0.23)	251 (0.22)
≥2 and <10	414 (0.36)	463 (0.41)
≥10	475 (0.41)	405 (0.36)
**Last hormonal birth control use within 5 years**		
Yes	303 (0.29)	272 (0.27)
No	743 (0.71)	725 (0.73)
**Parity at index age**		
0	282 (0.25)	278 (0.25)
1	209 (0.18)	187 (0.17)
2	415 (0.36)	395 (0.35)
≥3	244 (0.21)	259 (0.23)
**Age at first birth as categorical**		
Nulliparous	282 (0.25)	278 (0.25)
≤20	88 (0.08)	105 (0.09)
>20 and ≤25	242 (0.21)	259 (0.23)
>25 and ≤30	300 (0.26)	300 (0.27)
>30	238 (0.21)	177 (0.16)
**Last birth within 5 years of index age**		
Yes	103 (0.11)	76 (0.09)
No	802 (0.89)	791 (0.91)
**Breastfeeding among parous**		
Yes	728 (0.84)	697 (0.83)
No	140 (0.16)	144 (0.17)

Count (proportion) is shown for categorical variables and mean (standard deviation, SD) is shown for continuous variables.

Results for risk factor main effects and PRS-risk-factor interaction effects from CLR models based on non-Hispanic white families are shown in Table 3. Several risk factors were associated with young-onset breast cancer. The strongest risk factors were pre-menopausal status (odds ratio, OR = 1.85, 95% CI = 1.33, 2.58) and age at first birth, where a difference of 20 years corresponds to an OR of 2.19 (95% CI = 1.49, 3.87). Older age at menarche (OR = 0.93 for each year increase, 95% CI = 0.87, 0.99) and increased parity (OR = 0.9, per category 95% CI = 0.81, 1) were associated with reduced risk. As expected, higher PRS was associated with increased risk (OR = 2.33, 95% CI = 1.79, 3.04).

**Table 3:** Results of conditional logistic regression for either ductal carcinoma *in situ* or invasive breast cancer in non-Hispanic white families.

Variable Name	Variable Type	Risk factor model^a,b^		Interaction model^a,c^			
		OR (95% CI)	P	Risk factor OR (95% CI)	PRS OR (95% CI)^d^	Interaction ROR (95% CI)^e^	Interaction P
Ever Use Hormonal Birth Control	Binary	1.19 (0.89, 1.60)	0.25	1.31 (0.96, 1.80)	3.89 (2.16, 7.03)	0.56 (0.32, 1.00)	0.05
Hormonal Birth Control Use within 5 years	Binary	1.13 (0.90, 1.42)	0.28	1.15 (0.91, 1.45)	2.34 (1.72, 3.18)	0.85 (0.56, 1.29)	0.45
Last Birth within 5 years	Binary	1.37 (0.87, 2.16)	0.17	1.33 (0.84, 2.11)	2.15 (1.58, 2.92)	1.28 (0.66, 2.48)	0.46
Breastfeeding among Parous	Binary	1.11 (0.78, 1.57)	0.57	1.05 (0.74, 1.50)	1.53 (0.87, 2.70)	1.61 (0.89, 2.91)	0.11
Pre-menopause	Binary	1.85 (1.33, 2.58)	2 × 10^−4^	1.76 (1.26, 2.46)	1.23 (0.66, 2.30)	2.00 (1.07, 3.73)	0.03
Height (centered by the mean of controls’ heights, 65.0 inches)	Continuous	1.03 (0.98, 1.08)	0.19	1.03 (0.98, 1.08)	2.31 (1.77, 3.01)	1.03 (0.95, 1.11)	0.50
BMI at 30 s among Pre-menopausal	Continuous	0.98 (0.95, 1.01)	0.12	0.98 (0.95, 1.00)	1.52 (0.45, 5.14)	1.02 (0.97, 1.07)	0.48
Age at Menarche	Continuous	0.93 (0.87, 0.99)	0.03	0.92 (0.86, 0.99)	1.41 (0.29, 6.89)	1.04 (0.92, 1.18)	0.53
Age at First Birth among Parous	Continuous	1.04 (1.02, 1.07)	8 × 10^−4^	1.04 (1.02, 1.07)	1.64 (0.49, 5.42)	1.01 (0.97, 1.05)	0.6
Smoking: Former	Categorical	1.09 (0.87, 1.36)	0.28	1.09 (0.87, 1.36)	2.45 (1.82, 3.30)	0.90 (0.60, 1.34)	0.43
Current smoker		0.82 (0.58, 1.16)		0.84 (0.60, 1.19)		0.66 (0.35, 1.24)	
Alcohol	Categorical	0.83 (0.67, 1.03)	0.09	0.82 (0.67, 1.02)	2.07 (1.29, 3.33)	1.11 (0.75, 1.64)	0.58
Years Using Hormonal Birth Control	Categorical	1.06 (0.93, 1.20)	0.37	1.09 (0.96, 1.24)	3.27 (2.20, 4.86)	0.75 (0.59, 0.95)	0.02
Parity	Categorical	0.90 (0.81, 1.00)	0.05	0.90 (0.81, 1.00)	2.24 (1.54, 3.25)	1.02 (0.87, 1.21)	0.79
Age at First Birth	Categorical	1.05 (0.99, 1.12)	0.11	1.06 (0.99, 1.13)	2.75 (1.86, 4.04)	0.93 (0.82, 1.05)	0.25

^a^All models have birth order as a covariate. In addition, parity and age at first birth were adjusted for in breastfeeding models; alcohol and smoking were mutually adjusted for each other; age at menarche was adjusted for in models for years using birth control; and age at first birth was adjusted for in parity models.

^b^We assumed a linear trend in the risk factor effect in the risk-factor-only models for all categorical variables except for smoking.

^c^For all categorial variables, when testing for interaction effects, exposure was treated as categorical variable but a linear trend in the intraction term was assumed for all but the smoking variable. However, for the simplicity of presentation the risk-factor risk parameters shown above were based on a linear trend.

^d^OR is per unit change in PRS (Shi *et al*. 2017).

^e^The interaction parameter is the ratio of the PRS effect in the exposed category divided by the PRS effect in the unexposed category or those whose value of the risk factor is one unit less.

In interaction analyses, interactions between PRS and both ever use of hormonal birth control and years of use were evident for young-onset breast cancer. The positive association between PRS and breast cancer risk was lower for women who had ever (versus never) used hormonal birth control (ratio of odds ratios [ROR] = 0.56, 95% CI = 0.32, 1), where ROR is the ratio of odds ratios of PRS in the exposed group divided by that in the unexposed group. For example, the estimated OR of PRS in women who never used hormonal birth control was 3.89 per unit change, and that for the women who had ever used hormonal birth control was 2.18 ( = 3.89 * 0.56). The positive association between PRS and breast cancer risk decreased with each additional year of hormonal birth control use (ROR interaction=0.75, 95% CI = 0.59, 0.95). The association between PRS and risk of young-onset breast cancer was twice as strong for pre-menopausal woman as for post-menopausal women (ROR interaction=2.0, 95% CI = 1.07, 3.73). In contrast with that interaction with menopausal status, there was no evident interaction with age (interaction ROR = 1.0, 95% CI:(0.94, 1.07).

Results were similar when analyses were restricted to invasive breast cancer (Supplemental Table 2). Pre-menopausal status was still associated with increased risk (OR = 2.16, 95% CI = 1.49, 3.14), as was ever use of hormonal birth control (OR = 1.39, 95% CI = 1.01, 1.93). Alcohol use was associated with protection (OR = 0.8, 95% CI = 0.63, 1). Interactive effects of PRS with pre-menopausal status and hormonal birth control use largely remained the same. Analyses based on ER+ breast cancer families (Supplemental Table 3) and families from all ethnic groups (Supplemental Tables 4 and 5) showed similar results.

## Discussion

The nation-wide, family-based Two Sister Study was designed to study the roles of genetic and nongenetic risk factors in young-onset breast cancer. Although about 75% of breast cancers occur in women older than 50 and most published breast cancer GWAS have primarily included older cases, we previously showed that the multiplicative form of a 77-SNP PRS^16^ was also strongly related to breast cancer risk in young women^3^. Mavaddat *et al*. also found little evidence that the importance of the PRS for risk prediction is different for young onset^24^. In the current project, we assessed the multiplicative joint effects of 14 risk factors in conjunction with that PRS. Our interaction findings were largely null, though we did see evidence for effect-measure modification with menopausal status and with history of use of hormonal birth control. Based on our young-onset families, the estimated effect of PRS on breast cancer risk was approximately twice as strong in premenopausal versus postmenopausal women, and in women who had never used hormonal birth control versus those who had. Separate consideration of the reduced samples restricted to invasive or ER+ cancers did not reveal any important differences. It is worth noting that because we focus on multiplicative interactions in this study, we will miss identifying joint effects that are actually more than additive but not significantly different from multiplicative.

Rudolph *et al*.^17^ used the same 77-SNP PRS to carry out a large pooled analysis of gene-by-environment involving 20 studies participating in the Breast Cancer Association Consortium (which did not include the Sister Study or Two Sister Study). Their analyses were not focused on young-onset breast cancer, and they did not consider either menopausal status or history of use of hormonal contraception in their meta-analyses. They did report statistically-significant interactions between PRS and both height and alcohol use, where higher alcohol consumption and greater height were both associated with a reduction in the estimated effect of the PRS. The associations were particularly strong for ER+ breast cancer. We did not see compelling evidence for either interaction for the young-onset families in the Two Sister Study. The Rudolph *et al*. analyses also found some evidence that current post-menopausal users of combined menopausal hormone therapy (compared to never users) may have a stronger effect of PRS on ER+ breast cancer. We did not consider the interaction effects of hormone replacement therapy due to complications in our study design^19^ and the fact that the large majority of our women were premenopausal.

More recent analyses based on Breast Cancer Association Consortium have made it possible to include genetic variants with very small effects and have enabled the construction of complex polygenic risk scores that incorporate as many as 313 SNPs^24^, with some improvement in risk prediction. Future analyses of gene by environment interactions may benefit by using the more detailed genetic assessments, though the improvements may be modest.

The major limitations of the Two Sister Study are its modest sample size and its racially homogenous composition. For comparison, the pooled analysis conducted by Rudolph *et al*. included up to 23,000 breast cancer cases, versus our 1,291 young-onset cases. Ideally we would find a way to pool our results with other studies that include younger women, but because it is relatively rare, young-onset disease will always be a challenge to study. In terms of generalizability, our sample is predominantly non-Hispanic white women, and the results may not apply to other racial or ethnic groups. Also, our family-based design recruited cases within up to 4 years following their diagnosis, and retrospective studies can be subject to both survival bias and recall bias.

On the other hand, the family-based design is a notable strength of the Two Sister Study, as family-based methods offer robustness from bias due to population stratification when studying effects of genetic variants. Including risk factor information for both an affected and an unaffected offspring provided further protection from biases for assessment of gene-by-exposure interaction. Case-control studies and cohort studies are both vulnerable to bias in evaluating interaction because subpopulations can differ both in their exposure distribution and also in their genetic architecture (e.g., minor allele frequency and linkage disequilibrium patterns) in a way that can cause the true relative risks for marker alleles and exposures to covary across subpopulations^25^. These covariances can cause artifactual gene-by-environment interactions or can mask true associations. Family-based studies with exposure information from both an affected and an unaffected offspring should not be vulnerable to such distortion^18,25^.

In this analysis, we carried out a robust family-based analysis to investigate multiplicative interactions between a well-established PRS and nongenetic risk factors for young-onset breast cancer. Most of the measured interactions were compatible with the null, so that our data are consistent with the possibility that the joint effects of those factors and the PRS are multiplicative, but we observed evidence that both menopausal status and use of hormonal birth control modify the association between the PRS and young-onset breast cancer.

## Supplementary information

Supplementary information
