# Supplementary material for: Interactions between a Polygenic Risk Score and Non-genetic Risk Factors in Young-Onset Breast Cancer
Source: Sci Rep. 2020 Feb 24;10:3242. doi: 10.1038/s41598-020-60032-3 (PMC7039983; doi:10.1038/s41598-020-60032-3)
Supplement: Supplementary file 1 — Supplementary information [file 41598_2020_60032_MOESM1_ESM.docx]

**Interactions between a Polygenic Risk Score and Non-genetic Risk Factors**

**in Young-Onset Breast Cancer**

**Shi, M.^1^, O’Brien, K.M.^2^, Weinberg, C.R.^1^**

1. Biostatistics and Computational Biology Branch, National Institute of Environmental Health Sciences

2. Epidemiology Branch, National Institute of Environmental Health Sciences

Supplemental Table 1. Characteristics of all participants. Count (percentage) is shown for categorical variables and mean (standard deviation, SD) is shown for continuous variables.

| Variable | Case (N=1291) | Control (N=1259) |
| --- | --- | --- |
| **PRS mean (SD)** | 5.15 (0.46) | 5.03 (0.47) |
| **Age at Diagnosis mean (SD)** | 44.7 (4) |  |
| **Height mean (SD)** | 65.1 (2.6) | 64.9 (2.6) |
|  |  |  |
| **Race** |  |  |
| Non-Hispanic White | 1152 (0.89) |  |
| Non-Hispanic Black | 58 (0.04) |  |
| Hispanic | 46 (0.04) |  |
| Other | 35 (0.03) |  |
| **BMI at 30s among Pre-menopausal mean (SD)** | 23.6 (4) | 23.8 (4.4) |
| **Age at Menarche mean (SD)** | 12.7 (1.5) | 12.8 (1.5) |
| **Age at First Birth as Continuous mean (SD)** | 27.4 (5.4) | 26.6 (5.3) |
|  |  |  |
| **Invasive** |  |  |
| Yes | 1106 (0.86) |  |
| No | 185 (0.14) |  |
| **ER+** |  |  |
| Yes | 1016 (0.8) |  |
| No | 261 (0.2) |  |
| **Smoking** |  |  |
| Never | 848 (0.66) | 839 (0.67) |
| Former | 349 (0.27) | 307 (0.24) |
| Current | 94 (0.07) | 112 (0.09) |
| **Alcohol** |  |  |
| Never | 134 (0.1) | 107 (0.09) |
| <1 drink/day | 1011 (0.79) | 1006 (0.81) |
| ≥1 drink/day | 142 (0.11) | 128 (0.1) |
| **Pre-menopausal Status** | |  |
| Yes | 1195 (0.93) | 1128 (0.9) |
| No | 92 (0.07) | 130 (0.1) |
| **Birth Control Use** |  |  |
| Yes | 1167 (0.91) | 1125 (0.9) |
| No | 118 (0.09) | 131 (0.1) |
| **Years Using Birth Control** | |  |
| <2 | 289 (0.22) | 275 (0.22) |
| ≥2 and <10 | 464 (0.36) | 524 (0.42) |
| ≥10 | 532 (0.41) | 458 (0.36) |
| **Last Birth Control Use within 5 years** | | |
| Yes | 341 (0.29) | 308 (0.27) |
| No | 826 (0.71) | 817 (0.73) |
| **Parity** |  |  |
| 0 | 322 (0.25) | 321 (0.26) |
| 1 | 236 (0.18) | 224 (0.18) |
| 2 | 463 (0.36) | 434 (0.35) |
| ≥3 | 266 (0.21) | 278 (0.22) |
| **Age at First Birth as Categorical** | |  |
| Nulliparous | 322 (0.25) | 321 (0.26) |
| ≤20 | 107 (0.08) | 126 (0.1) |
| >20 and ≤25 | 263 (0.2) | 282 (0.22) |
| >25 and ≤30 | 339 (0.26) | 327 (0.26) |
| >30 | 256 (0.2) | 201 (0.16) |
| **Last Birth within 5 years** |  |  |
| Yes | 113 (0.11) | 88 (0.09) |
| No | 895 (0.89) | 878 (0.91) |
| **Breastfeeding among Parous** | |  |
| Yes | 800 (0.83) | 765 (0.82) |
| No | 165 (0.17) | 171 (0.18) |

Supplement Table 2. Results of conditional logistic regression for invasive breast cancer in non-Hispanic white families

|  |  | Risk factor only model^a,b^ | |  | Interaction model^a,c^ | | | |
| --- | --- | --- | --- | --- | --- | --- | --- | --- |
| Variable Name | Variable Type | OR (95% CI) | P |  | Risk factor OR  (95% CI) | PRS OR (95% CI)^d^ | Interaction ROR (95% CI)^e^ | Interaction P |
| Ever Use Hormonal Birth Control | Binary | 1.39 (1.01,1.93) | 0.04 |  | 1.50 (1.06,2.11) | 3.69 (1.94,7.04) | 0.60 (0.32,1.13) | 0.11 |
| Hormonal Birth Control Use within 5 years | Binary | 1.11 (0.88,1.41) | 0.38 |  | 1.13 (0.89,1.44) | 2.35 (1.68,3.28) | 0.86 (0.55,1.34) | 0.5 |
| Last Term Birth within 5 years | Binary | 1.28 (0.78,2.09) | 0.33 |  | 1.22 (0.74,2.02) | 2.13 (1.53,2.98) | 1.48 (0.71,3.08) | 0.29 |
| Breastfeeding among Parous | Binary | 1.05 (0.72,1.53) | 0.8 |  | 1.01 (0.69,1.49) | 1.75 (0.96,3.20) | 1.42 (0.75,2.66) | 0.28 |
| Pre-menopause | Binary | 2.16 (1.49,3.14) | 3x10^-5^ |  | 2.08 (1.43,3.04) | 1.07 (0.54,2.14) | 2.35 (1.18,4.69) | 0.01 |
| Height (centered by the mean of controls’ heights, 65.0 inches) | Continuous | 1.03 (0.98,1.09) | 0.22 |  | 1.03 (0.98,1.09) | 2.34 (1.76,3.12) | 1.00 (0.92,1.09) | 0.93 |
| BMI at 30s among Pre-menopausal | Continuous | 0.98 (0.95,1.01) | 0.1 |  | 0.97 (0.95,1.00) | 1.61 (0.44,5.97) | 1.02 (0.96,1.07) | 0.57 |
| Age at Menarche | Continuous | 0.92 (0.86,0.99) | 0.03 |  | 0.92 (0.86,0.99) | 1.61 (0.29,8.79) | 1.03 (0.90,1.17) | 0.66 |
| Age at First Term Birth among Parous | Continuous | 1.04 (1.01,1.07) | 0.003 |  | 1.04 (1.01,1.07) | 1.66 (0.45,6.10) | 1.01 (0.97,1.06) | 0.63 |
| Smoking: Former | Categorical | 1.16 (0.91,1.47) | 0.36 |  | 1.15 (0.90,1.47) | 2.40 (1.73,3.31) | 0.95 (0.61,1.46) | 0.79 |
| Current smoker |  | 0.92 (0.64,1.33) |  |  | 0.94 (0.65,1.35) |  | 0.79 (0.41,1.53) |  |
| Alcohol | Categorical | 0.80 (0.63,1.00) | 0.05 |  | 0.79 (0.63,1.00) | 2.05 (1.23,3.43) | 1.13 (0.74,1.72) | 0.57 |
| Years Using Hormonal Birth Control | Categorical | 1.09 (0.96,1.25) | 0.19 |  | 1.12 (0.97,1.29) | 3.18 (2.06,4.91) | 0.77 (0.59,1.01) | 0.06 |
| Parity | Categorical | 0.94 (0.83,1.05) | 0.26 |  | 0.93 (0.83,1.05) | 2.30 (1.53,3.46) | 1.01 (0.85,1.21) | 0.91 |
| Age at First Term Birth | Categorical | 1.06 (0.99,1.14) | 0.11 |  | 1.07 (0.99,1.14) | 2.74 (1.80,4.15) | 0.93 (0.81,1.07) | 0.32 |

^a^ All models have birth order as a covariate. In addition, parity and age at first birth were adjusted for in breastfeeding models; alcohol and smoking was mutually adjusted for each other; age at menarche was adjusted for in models for years using birth control; and age at first birth was adjusted for in parity models.

^b^ We assumed a linear trend in the risk factor effect in the risk-factor-only models for all categorical variables except for smoking

^c^ For all categorial variables, when testing for interaction effects, the risk factor was treated as categorical variable but a linear trend in the interaction term was assumed for all but the smoking variable. However, for the simplicity of presentation the risk-factor risk parameters shown above were based on a linear trend.

^d^ OR is per unit change in PRS (Shi et al. 2017)

^e^ The interaction parameter is the ratio of the PRS effect in the exposed category divided by the PRS effect in the unexposed category or those whose value of the risk factor is one unit less.

Supplemental Table 3. Results of conditional logistic regression in non-Hispanic white ER+ breast cancer families

|  |  | Exposure only model^a,b^ | |  | Interaction model^a,c^ | | | |
| --- | --- | --- | --- | --- | --- | --- | --- | --- |
| Variable Name | Variable Type | OR (95% CI) | P |  | Exposure OR (95% CI) | PRS OR (95% CI)^d^ | Interaction ROR (95% CI)^e^ | Interaction P |
| Ever Use Birth Control | Binary | 1 (0.72,1.39) | 1 |  | 1.06 (0.74,1.53) | 2.92 (1.61,5.31) | 0.78 (0.44,1.41) | 0.41 |
| Hormonal Birth Control Use within 5 years | Binary | 1.1 (0.85,1.42) | 0.46 |  | 1.15 (0.88,1.5) | 2.51 (1.82,3.45) | 0.77 (0.5,1.18) | 0.23 |
| Last Birth < 5 years | Binary | 1.26 (0.74,2.12) | 0.39 |  | 1.27 (0.74,2.16) | 2.26 (1.65,3.08) | 0.93 (0.47,1.83) | 0.84 |
| Breastfeeding among Parous | Binary | 1.08 (0.74,1.58) | 0.7 |  | 1.01 (0.69,1.5) | 1.55 (0.87,2.78) | 1.6 (0.88,2.9) | 0.12 |
| Pre-menopause | Binary | 2.1 (1.44,3.07) | 8x10^-5^ |  | 1.93 (1.31,2.84) | 1.36 (0.72,2.58) | 1.83 (0.97,3.47) | 0.06 |
| Height (centered by the mean of controls’ heights, 65.0 inches) | Continuous | 1.03 (0.98,1.09) | 0.27 |  | 1.03 (0.97,1.09) | 2.35 (1.79,3.09) | 1.02 (0.94,1.1) | 0.64 |
| BMI at 30s among Pre-menopausal | Continuous | 0.98 (0.95,1.01) | 0.2 |  | 0.98 (0.95,1.01) | 2.58 (0.75,8.9) | 1 (0.95,1.05) | 0.91 |
| Age at Menarche | Continuous | 0.94 (0.88,1.02) | 0.14 |  | 0.94 (0.87,1.02) | 2.24 (0.46,10.98) | 1 (0.89,1.14) | 0.95 |
| Age at First Birth among Parous | Continuous | 1.04 (1.02,1.07) | 0.002 |  | 1.04 (1.01,1.07) | 1.47 (0.45,4.8) | 1.02 (0.97,1.06) | 0.47 |
| Smoking: Former smoker | Categorical | 1.08 (0.84,1.39) | 0.74 |  | 1.1 (0.85,1.42) | 2.45 (1.8,3.33) | 0.89 (0.59,1.33) | 0.72 |
| Smoking: Current smoker | Categorical | 0.96 (0.65,1.42) |  |  | 0.99 (0.66,1.48) | 2.45 (1.8,3.33) | 0.79 (0.41,1.51) |  |
| Alcohol | Categorical | 0.78 (0.61,0.98) | 0.03 |  | 0.76 (0.59,0.97) | 1.96 (1.21,3.15) | 1.19 (0.8,1.78) | 0.37 |
| Years Using Birth Control | Categorical | 1.01 (0.88,1.16) | 0.88 |  | 1.05 (0.91,1.22) | 3.06 (2.04,4.57) | 0.8 (0.62,1.02) | 0.08 |
| Parity | Categorical | 0.9 (0.8,1.01) | 0.07 |  | 0.9 (0.79,1.01) | 2.33 (1.59,3.43) | 1.01 (0.85,1.19) | 0.94 |
| Age at First Birth | Categorical | 1.06 (0.98,1.14) | 0.12 |  | 1.06 (0.99,1.14) | 2.48 (1.66,3.68) | 0.98 (0.86,1.11) | 0.79 |

^a^ All models have birth order as a covariate. In addition, parity and age at first birth were adjusted for in breastfeeding models; alcohol and smoking was mutually adjusted for each other; age at menarche was adjusted for in models for years using birth control; and age at first birth was adjusted for in parity models.

^b^ We assumed a linear trend in the exposure effect in the exposure-only models for all categorical variables except for smoking

^c^ For all categorial variables, when testing for interaction effects, exposure was treated as categorical variable but a linear trend in the interaction term was assumed for all but the smoking variable. However, for the simplicity of presentation the exposure risk parameters shown above were based on a linear trend.

^d^ OR is per unit change in PRS (Shi et al. 2017)

^e^ The interaction parameter is the ratio of the PRS effect in the exposed category divided by the PRS effect in the unexposued category or those whose value of the risk factor is one unit less.

Supplemental Table 4. Results of conditional logistic regression based on all breast cancer families

|  |  | Exposure only model^a,b^ | |  | Interaction model^a,c^ | | | |
| --- | --- | --- | --- | --- | --- | --- | --- | --- |
| Variable Name | Variable Type | OR (95% CI) | P |  | Exposure OR (95% CI) | PRS OR (95% CI)^d^ | Interaction ROR (95% CI)^e^ | Interaction P |
| Ever Use Hormonal Birth Control | Binary | 1.12 (0.85,1.47) | 0.43 |  | 1.22 (0.91,1.64) | 3.58 (2.07,6.2) | 0.61 (0.36,1.03) | 0.06 |
| Hormonal Birth Control Use within 5 years | Binary | 1.15 (0.93,1.41) | 0.2 |  | 1.18 (0.95,1.47) | 2.47 (1.84,3.31) | 0.8 (0.55,1.17) | 0.25 |
| Last Term Birth < 5 years | Binary | 1.25 (0.82,1.90) | 0.29 |  | 1.22 (0.8,1.85) | 2.07 (1.54,2.77) | 1.32 (0.71,2.45) | 0.37 |
| Breastfeeding among Parous | Binary | 1.09 (0.79,1.51) | 0.6 |  | 1.05 (0.76,1.46) | 1.65 (0.97,2.81) | 1.38 (0.8,2.38) | 0.25 |
| Pre-menopause | Binary | 1.73 (1.27,2.36) | 5x10^-4^ |  | 1.65 (1.2,2.27) | 1.47 (0.81,2.65) | 1.62 (0.9,2.9) | 0.11 |
| Height (centered by the mean of controls’ heights, 64.9 inches) | Continuous | 1.03 (0.99,1.08) | 0.14 |  | 1.03 (0.99,1.08) | 2.28 (1.77,2.93) | 1.01 (0.94,1.09) | 0.72 |
| BMI at 30s among Pre-menopausal | Continuous | 0.98 (0.96,1.01) | 0.2 |  | 0.98 (0.95,1.01) | 1.38 (0.45,4.24) | 1.02 (0.98,1.07) | 0.35 |
| Age at Menarche | Continuous | 0.93 (0.87,0.99) | 0.02 |  | 0.93 (0.87,0.99) | 1.71 (0.38,7.59) | 1.02 (0.91,1.15) | 0.7 |
| Age at First Term Birth among Parous | Continuous | 1.04 (1.02,1.07) | 5x10^-4^ |  | 1.04 (1.02,1.06) | 0.92 (0.31,2.69) | 1.03 (0.99,1.07) | 0.11 |
| Smoking: Former | Categorical | 1.13 (0.91,1.39) | 0.21 |  | 1.13 (0.91,1.4) | 2.38 (1.8,3.15) | 0.93 (0.64,1.35) | 0.45 |
| Current smoker |  | 0.85 (0.62,1.18) |  |  | 0.88 (0.63,1.22) | 2.38 (1.8,3.15) | 0.68 (0.38,1.24) |  |
| Alcohol | Categorical | 0.87 (0.72,1.07) | 0.19 |  | 0.85 (0.7,1.05) | 1.83 (1.18,2.86) | 1.23 (0.85,1.78) | 0.23 |
| Years Using Hormonal Birth Control | Categorical | 1.05 (0.93,1.18) | 0.41 |  | 1.08 (0.96,1.22) | 2.94 (2.03,4.25) | 0.8 (0.64,1.01) | 0.06 |
| Parity | Categorical | 0.93 (0.84,1.03) | 0.15 |  | 0.93 (0.84,1.03) | 2.35 (1.65,3.34) | 0.98 (0.84,1.15) | 0.84 |
| Age at First Term Birth | Categorical | 1.05 (0.99,1.11) | 0.12 |  | 1.06 (0.99,1.12) | 2.66 (1.87,3.8) | 0.93 (0.83,1.04) | 0.24 |

^a^ All models have birth order as a covariate. In addition, parity and age at first birth were adjusted for in breastfeeding models; alcohol and smoking was mutually adjusted for each other; age at menarche was adjusted for in models for years using birth control; and age at first birth was adjusted for in parity models.

^b^ We assumed a linear trend in the exposure effect in the exposure-only models for all categorical variables except for smoking

^c^ For all categorial variables, when testing for interaction effects, exposure was treated as categorical variable but a linear trend in the interaction term was assumed for all but the smoking variable. However, for the simplicity of presentation the risk factor risk parameters shown above were based on a linear trend.

^d^ OR is per unit change in PRS (Shi et al. 2017)

^e^ The interaction parameter is the ratio of the PRS effect in the exposed category divided by the PRS effect in the unexposed category or those whose value of the risk factor is one unit less.

Supplemental Table 5. Results of conditional logistic regression for invasive breast cancer using all families

|  |  | Exposure only model^a,b^ | |  | Interaction model^a,c^ | | | |
| --- | --- | --- | --- | --- | --- | --- | --- | --- |
| Variable Name | Variable Type | OR (95% CI) | P |  | Exposure OR (95% CI) | PRS OR (95% CI)^e^ | Interaction ROR (95% CI)^e^ | Interaction P |
| Ever Use Hormonal Birth Control | Binary | 1.22 (0.90,1.66) | 0.19 |  | 1.31 (0.95,1.8) | 3.31 (1.84,5.97) | 0.65 (0.37,1.16) | 0.14 |
| Hormonal Birth Control Use within 5 years | Binary | 1.13 (0.90,1.41) | 0.29 |  | 1.16 (0.92,1.46) | 2.44 (1.78,3.36) | 0.82 (0.55,1.23) | 0.34 |
| Last Term Birth within 5 years | Binary | 1.14 (0.73,1.79) | 0.55 |  | 1.11 (0.7,1.74) | 2.02 (1.48,2.77) | 1.46 (0.74,2.86) | 0.27 |
| Breastfeeding among Parous | Binary | 1.07 (0.76,1.52) | 0.69 |  | 1.05 (0.74,1.5) | 1.9 (1.08,3.34) | 1.17 (0.65,2.08) | 0.6 |
| Pre-menopause | Binary | 1.95 (1.38,2.76) | 1x10^-4^ |  | 1.86 (1.31,2.64) | 1.37 (0.72,2.6) | 1.74 (0.92,3.30) | 0.09 |
| Height (centered by the mean of controls’ heights, 64.9 inches) | Continuous | 1.04 (0.99,1.09) | 0.17 |  | 1.04 (0.99,1.09) | 2.28 (1.74,2.99) | 0.99 (0.92,1.07) | 0.82 |
| BMI at 30s among Pre-menopausal | Continuous | 0.98 (0.96,1.01) | 0.21 |  | 0.98 (0.95,1.01) | 1.28 (0.38,4.27) | 1.03 (0.98,1.08) | 0.32 |
| Age at Menarche | Continuous | 0.92 (0.86,0.98) | 0.01 |  | 0.92 (0.85,0.98) | 1.98 (0.4,9.67) | 1.01 (0.89,1.14) | 0.86 |
| Age at First Term Birth among Parous | Continuous | 1.04 (1.02,1.07) | 0.001 |  | 1.04 (1.01,1.07) | 0.79 (0.25,2.49) | 1.04 (1,1.08) | 0.08 |
| Smoking: Former | Categorical | 1.19 (0.95,1.50) | 0.24 |  | 1.19 (0.94,1.5) | 2.31 (1.7,3.13) | 0.97 (0.65,1.45) | 0.76 |
| Current smoker |  | 0.95 (0.67,1.34) |  |  | 0.96 (0.68,1.37) | 2.31 (1.7,3.13) | 0.8 (0.43,1.47) |  |
| Alcohol | Categorical | 0.84 (0.68,1.04) | 0.1 |  | 0.82 (0.66,1.02) | 1.78 (1.1,2.87) | 1.26 (0.85,1.87) | 0.22 |
| Years Using Hormonal Birth Control | Categorical | 1.07 (0.94,1.22) | 0.29 |  | 1.09 (0.96,1.24) | 2.78 (1.87,4.13) | 0.84 (0.66,1.07) | 0.16 |
| Parity | Categorical | 0.97 (0.87,1.08) | 0.54 |  | 0.97 (0.87,1.09) | 2.46 (1.68,3.6) | 0.95 (0.81,1.12) | 0.58 |
| Age at First Term Birth | Categorical | 1.06 (0.99,1.13) | 0.1 |  | 1.07 (1,1.14) | 2.68 (1.83,3.91) | 0.93 (0.82,1.05) | 0.23 |

^a^ All models have birth order as a covariate. In addition, parity and age at first birth were adjusted for in breastfeeding models; alcohol and smoking was mutually adjusted for each other; age at menarche was adjusted for in models for years using birth control; and age at first birth was adjusted for in parity models.

^b^ We assumed a linear trend in the exposure effect in the exposure-only models for all categorical variables except for smoking

^c^ For all categorial variables, when testing for interaction effects, exposure was treated as categorical variable but a linear trend in the interaction term was assumed for all but the smoking variable. However, for the simplicity of presentation the risk factor risk parameters shown above were based on a linear trend.

^d^ OR is per unit change in PRS (Shi et al. 2017)

^e^ The interaction parameter is the ratio of the PRS effect in the exposed category divided by the PRS effect in the unexposed category or those whose value of the risk factor is one unit less.
